# The human resource for health situation in Zambia: deficit and maldistribution

**DOI:** 10.1186/1478-4491-9-30

**Published:** 2011-12-19

**Authors:** Paulo Ferrinho, Seter Siziya, Fastone Goma, Gilles Dussault

**Affiliations:** 1 International Public Health and Biostatistics Unit, CMDT, Instituto de Higiene e Medicina Tropical, Universidade Nova de Lisboa and Associação para o Desenvolvimento e Cooperação Garcia de Orta, Lisbon, Portugal; 2 School of Medicine, University of Zambia, Lusaka, Zambia

## Abstract

**Introduction:**

Current health policy directions in Zambia are formulated in the National Health Strategic Plan. The Plan focuses on national health priorities, which include the human resources (HR) crisis. In this paper we describe the way the HRH establishment is distributed in the different provinces of Zambia, with a view to assess the dimension of shortages and of imbalances in the distribution of health workers by province and by level of care.

**Population and methods:**

We used secondary data from the "March 2008 payroll data base", which lists all the public servants on the payroll of the Ministry of Health and of the National Health Service facilities. We computed rates and ratios and compared them.

**Results:**

The highest relative concentration of all categories of workers was observed in Northern, Eastern, Lusaka, Western and Luapula provinces (in decreasing order of number of health workers).

The ratio of clinical officers (mid-level clinical practitioners) to general medical officer (doctors with university training) varied from 3.77 in the Lusaka to 19.33 in the Northwestern provinces. For registered nurses (3 to 4 years of mid-level training), the ratio went from 3.54 in the Western to 15.00 in Eastern provinces and for enrolled nurses (two years of basic training) from 4.91 in the Luapula to 36.18 in the Southern provinces.

This unequal distribution was reflected in the ratio of population per cadre. The provincial distribution of personnel showed a skewed staff distribution in favour of urbanized provinces, e.g. in Lusaka's doctor: population ratio was 1: 6,247 compared to Northern Province's ratio of 1: 65,763.

In the whole country, the data set showed only 109 staff in health posts: 1 clinical officer, 3 environmental health technologists, 2 registered nurses, 12 enrolled midwives, 32 enrolled nurses, and 59 other.

The vacancy rates for level 3 facilities(central hospitals, national level) varied from 5% in Lusaka to 38% in Copperbelt Province; for level 2 facilities (provincial level hospitals), from 30% for Western to 70% for Copperbelt Province; for level 1 facilities (district level hospitals), from 54% for the Southern to 80% for the Western provinces; for rural health centres, vacancies varied from 15% to 63% (for Lusaka and Luapula provinces respectively); for urban health centres the observed vacancy rates varied from 13% for the Lusaka to 96% for the Western provinces. We observed significant shortages in most staff categories, except for support staff, which had a significant surplus.

**Discussion and Conclusions:**

This case study documents how a peaceful, politically stable African country with a longstanding tradition of strategic management of the health sector and with a track record of innovative approaches dealt with its HRH problems, but still remains with a major absolute and relative shortage of health workers. The case of Zambia reinforces the idea that training more staff is necessary to address the human resources crisis, but it is not sufficient and has to be completed with measures to mitigate attrition and to increase productivity.

## Introduction

This case study documents how a peaceful, politically stable African country with a longstanding tradition of strategic management of the health sector and with a track record of innovative approaches, dealt with its health workforce problems, but still faces a major absolute and relative deficit of health workers. We briefly describe the country context and we use official data from 2008 to analyse various dimensions of the health workforce, such as vacancies, attrition, and geographical imbalances. The case of Zambia reinforces the idea that training more staff is necessary to address the human resources crisis, but it is not sufficient and has to be completed with measures to mitigate attrition and to increase productivity.

## General Background

Zambia's population was estimated at just under 12 million in 2007 by the United Nations. The country is divided into 9 provinces and 72 districts. It has one of the lowest Human Development Index (0.481, ranking 164 in the world), the second lowest for Southern Africa, after Mozambique (http://www.pnud.org.br/pobreza_desigualdade/reportagens/index.php?id01=3324&lay=pde, accessed on 3 August 2009). It has one of the highest prevalence rates for HIV/AIDS in Africa (15.2%) (http://www.who.int/gho/countries/zmb/country_profiles/en/index.html, accessed on 17 January 2011).

Current policy directions are formulated in the National Health Strategic Plan (NHSP 2006-2010) [1], the fourth of its kind. It presents a major departure from previous plans, in that it establishes national health priorities, which include addressing the human resources for health (HRH) crisis [2,3]. The recognition of HRH as a priority derives from the estimation by the Ministry of Health (MoH) that health services function with less than half of the health workers required to deliver basic health services [4].

In addition to the national health service (NHS) facilities, there is an emerging urban private-for-profit sector, plus private mine-based hospitals, and a not-for-profit private sector working in close partnership with the public services. At the time of the study, of the 1327 healthcare facilities in Zambia, 85% are government run facilities, 9% are private sector facilities and 6% are religious affiliated facilities. Most (99%) of urban households reside within 5 km of a health facility compared to 50% of rural households [5].

There are six levels of care in the public sector and corresponding facilities (outreach services, health posts, health centres, and level 1- district, level-2 provincial and level-3 central hospitals).

Health Posts are intended to cater for populations of 500 households (3,500 people) in rural areas and 1,000 households (7,000 people) in the urban areas, or to be established within a 5 Km radius for sparsely populated areas. The target is 3,000 health posts. In 2008, there were 171 health posts. They offer basic first aid rather than curative care.

Health Centres include Urban Health Centres, which are intended to serve a catchment population of 30,000 to 50,000 people, and Rural Health Centres, servicing a catchment area of 29 Km radius or a population of 10,000. The target is 1,385. Totals of 1029 rural health centres and 265 urban health centres were recorded in 2008. For the purpose of defining approved prototype staff establishments, health centres are further subdivided into large and medium urban, zonal and medium rural [6,7].

1^st ^Level Referral Hospitals are found in 60 of the 72 districts and are intended to serve a population of between 80,000 and 200,000 with medical, surgical, obstetric and diagnostic services, including all clinical services to support health centre referrals. Currently, there are 72 1^st ^Level Referral Hospitals. There is an approved prototype staff establishment of 192 workers, common to all 1^st ^level hospitals [6,7].

2^nd ^level referral, Provincial or General Hospitals are 2^nd ^level hospitals at provincial level and are intended to cater for a catchment population of 200,000 to 800,000 people, with services in internal medicine, general surgery, paediatrics, obstetrics and gynaecology, dental, psychiatry and intensive care services. There are 21 level 2 hospitals. These hospitals are also planned to act as referral centres for the 1^st ^level institutions, including the provision of technical back-up and training functions. There is need to rationalize the distribution of these facilities through right-sizing. For the purpose of defining approved prototype staff establishments, 2^nd ^level hospitals are further subdivided into urban (with a staff establishment of 629) and rural (with a staff establishment of 384) [6,7].

3^rd ^level or Central Hospitals are for catchment populations of 800,000 and above, and have sub-specializations in internal medicine, surgery, paediatrics, obstetrics, gynaecology, intensive care, psychiatry, training and research. These hospitals also act as referral centres for 2^nd ^level hospitals. Currently there are 6 such facilities in the country, of which 3 are in the Copperbelt Province. Again there is need to rationalize the distribution of these facilities [6,7].

Contractual arrangements with private providers, particularly the mission and mining sectors, are common [6,7].

The National Health Service staff establishment covers these six types of facilities [8]. In this paper we describe the way this establishment is distributed in the different provinces of Zambia.

## The Health Workforce: Stock and Distribution

### Population and methods

Using the "March 2008 payroll data base", that lists all public servants on the payroll of MoH and of NHS facilities, we analysed data on the distribution of health workers by category and post, province, type of health facility and health care level. Figures on the number of inhabitants were obtained from the Zambia 2000 "census of population and housing", and extrapolated using expected growth rates for each province. Population figures for district level were not available.

The results of this analysis are explained in light of the literature available, and of findings from in-depth interviews by three of the authors (PF, SS & FG) with key informants and personal observations carried out in the context of another parallel study (P Ferrinho, M Sidat, F Goma, G Dussault: Task-shifting - opinions and experiences of health workers in the Mozambican and Zambian National Health Services, submitted to Human Resour Health 2011).

### Results

#### Distribution of personnel across provinces

The most numerous categories of health workers in all provinces are the Zambia Enrolled nurses, followed by Zambia Enrolled Midwives and Registered nurses. Variations between provinces observed at the level of health specific cadres are greater than at that of general support staff. The highest concentrations of health specific cadres are observed, in decreasing order, in the Central, Southern, Copperbelt and Northwestern provinces (Table 1).

**Table 1:** Distribution of health workers (% of total workers on NHS payroll) by province

Occupation	Central	Copperbelt	Eastern	Luapula	Lusaka	North-Western	Northern	Southern	Western
	
	Total = 1787	Total = 4987	Total = 2281	Total = 1123	Total = 5617	Total = 1190	Total = 2153	Total = 3533	Total = 1423
	
	Percent	Percent	Percent	Percent	Percent	Percent	Percent	Percent	Percent
**General Medical Officer**	0,62	0,62	0,26	0,98	1,09	0,25	0,42	0,31	0,91

**Anaesthesiologist**	0,00	0,00	0,00	0,09	0,00	0,00	0,00	0,00	0,00

**Biomedical Scientist**	0,06	0,10	0,00	0,00	0,37	0,00	0,05	0,11	0,00

**Clinical Officer**	5,99	4,31	4,12	4,36	4,09	4,87	4,37	5,26	5,41

**Clinical Officer Anaesthesia**	0,28	0,14	0,13	0,27	0,18	0,25	0,23	0,37	0,07

**Clinical Officer Dental**	0,11	0,04	0,04	0,00	0,07	0,00	0,00	0,08	0,07

**Clinical Officer Dermatology**	0,06	0,04	0,00	0,00	0,00	0,17	0,05	0,00	0,07

**Clinical Officer Eye, Nose, and Throat**	0,00	0,02	0,00	0,00	0,00	0,00	0,00	0,00	0,00

**Clinical Officer Ophthalmology**	0,00	0,04	0,04	0,00	0,05	0,08	0,05	0,06	0,28

**Clinical Officer TB, HIV/AIDS**	0,00	0,00	0,00	0,00	0,00	0,00	0,00	0,03	0,00

**Clinical Officer Psychiatry**	0,11	0,04	0,26	0,00	0,05	0,00	0,05	0,08	0,00

**Consultant**	0,11	0,28	0,13	0,18	0,84	0,00	0,00	0,06	0,07

**Consultant Anaesthesia**	0,00	0,02	0,00	0,00	0,00	0,08	0,00	0,00	0,00

**Consultant Obstetrics and Gynaecologist**	0,06	0,00	0,00	0,09	0,00	0,08	0,00	0,03	0,00

**Consultant Ophthalmology**	0,00	0,02	0,00	0,00	0,00	0,00	0,00	0,00	0,00

**Consultant Surgeon**	0,00	0,02	0,00	0,00	0,00	0,00	0,00	0,00	0,00

**Dental Surgeon**	0,06	0,06	0,04	0,00	0,07	0,08	0,05	0,00	0,07

**Dental Technician**	0,06	0,00	0,00	0,00	0,00	0,00	0,00	0,00	0,00

**Dental Technologist**	0,11	0,12	0,22	0,09	0,05	0,08	0,05	0,14	0,07

**Dental Therapist**	0,78	0,58	0,44	0,36	0,61	0,34	0,37	0,48	0,49

**Environmental Health Expert**	0,22	0,22	0,13	0,36	0,05	0,50	0,28	0,23	0,07

**Environmental Health Officer**	0,22	0,12	0,35	0,45	0,14	0,17	0,42	0,23	0,35

**Environmental Health Technologist**	4,92	1,82	4,56	3,74	1,51	4,37	3,99	4,70	6,25

**Laboratory Technologist**	1,12	0,94	0,53	0,98	0,66	0,76	0,56	0,76	0,63

**Laboratory Technician**	0,95	1,16	0,66	0,98	0,64	0,84	0,65	0,82	0,70

**Medical Licentiate**	0,11	0,02	0,26	0,18	0,07	0,08	0,14	0,03	0,21

**Nutritionist**	0,28	0,18	0,39	0,18	0,28	0,17	0,23	0,25	0,14

**Occupational Health Technologist**	0,00	0,00	0,00	0,00	0,00	0,00	0,00	0,03	0,00

**Occupational Therapist**	0,00	0,00	0,00	0,00	0,00	0,00	0,00	0,03	0,00

**Pharmacist**	0,28	0,24	0,13	0,09	0,16	0,00	0,14	0,20	0,14

**Pharmacy Dispenser**	1,68	1,46	0,79	0,71	1,10	0,50	0,74	1,25	1,48

**Pharmacy Technician**	0,11	0,12	0,04	0,27	0,00	0,17	0,05	0,03	0,14

**Pharmacy Technologist**	0,28	0,42	0,53	0,53	0,39	0,34	0,19	0,42	0,14

**Physiotherapist**	0,95	0,80	0,35	0,45	0,62	0,42	0,37	0,57	0,56

**Physiotherapy Technologist**	0,06	0,04	0,18	0,27	0,04	0,00	0,14	0,20	0,07

**Public Health Nurse**	0,11	0,04	0,04	0,00	0,14	0,08	0,05	0,00	0,21

**Radiographer**	0,78	0,78	0,53	0,36	0,64	0,34	0,46	0,79	0,07

**Radiography Technologist**	0,00	0,00	0,00	0,00	0,07	0,00	0,19	0,00	0,00

**Registered ICU Nurse**	0,00	0,00	0,04	0,00	0,00	0,00	0,00	0,03	0,00

**Registered Midwife**	2,13	1,95	0,70	0,53	1,80	1,18	0,74	1,33	0,84

**Registered Nurse**	5,60	6,36	3,95	4,81	6,48	3,36	5,57	4,02	3,23

**Registered Psychiatry Nurse**	0,11	0,02	0,00	0,00	0,27	0,00	0,00	0,06	0,00

**Registered Theatre Nurse**	0,28	0,22	0,22	0,36	0,32	0,08	0,19	0,20	0,14

**Registrar**	0,06	0,48	0,04	0,27	0,80	0,00	0,00	0,14	0,00

**Registered Nurse Ophthalmology**	0,00	0,00	0,00	0,00	0,02	0,00	0,00	0,00	0,00

**Resident Medical Officer**	0,73	1,44	0,70	0,80	1,57	1,26	0,65	1,02	0,77

**Zambia Enrolled Midwife**	9,85	7,62	7,85	4,81	5,38	3,70	6,60	11,27	5,55

**Zambia Enrolled Nurse**	31,45	20,93	20,12	24,93	18,23	29,16	16,40	22,90	22,98

**Zambia Enrolled Ophthalmology**	0,00	0,00	0,00	0,00	0,00	0,00	0,00	0,03	0,00

**Zambia Enrolled Psychiatry Nurse**	0,22	0,16	0,09	0,45	0,73	0,00	0,05	0,03	0,14

**Zambia Enrolled Theatre Nurse**	0,17	0,14	0,39	0,18	0,05	0,00	0,19	0,14	0,21

**Other**	28,93	45,84	50,72	46,93	50,35	46,22	55,36	41,30	47,43

Source: MoH, March 2008 payroll data base

Ratios of clinical officers, who are mid-level practitioners to general medical officer, who are physicians with university training, varied from 3.77 in Lusaka to 19.33 in Northwestern Province. For registered nurses (3 to 4 years of mid-level training), the ratio varied from 3.54 in Western to 15.00 in Eastern Province, and for Zambia enrolled nurses (two years of basic training) from 25.15 in Western Province to 115.67 in the Northwestern Province. The highest ratios for health specific cadres are observed for Zambia Enrolled nurses, followed by Zambia Enrolled Midwives and Registered nurses (Table 2).

**Table 2:** Ratio of different occupations per General Medical Officer per province

Occupation	Central	Copperbelt	Eastern	Luapula	Lusaka	North-Western	Northern	Southern	Western
	
	ratio	ratio	ratio	ratio	ratio	ratio	ratio	ratio	ratio
**General Medical Officer**	1,00	1,00	1,00	1,00	1,00	1,00	1,00	1,00	1,00

**Anaesthesiologist**	0,00	0,00	0,00	0,09	0,00	0,00	0,00	0,00	0,00

**Biomedical Scientist**	0,09	0,16	0,00	0,00	0,34	0,00	0,11	0,36	0,00

**Clinical Officer**	9,73	6,94	15,67	4,45	3,77	19,33	10,44	16,91	5,92

**Clinical Officer Anaesthesia**	0,45	0,23	0,50	0,27	0,16	1,00	0,56	1,18	0,08

**Clinical Officer Dental**	0,18	0,06	0,17	0,00	0,07	0,00	0,00	0,27	0,08

**Clinical Officer Dermatology**	0,09	0,06	0,00	0,00	0,00	0,67	0,11	0,00	0,08

**Clinical Officer Eye, Nose, and Throat**	0,00	0,03	0,00	0,00	0,00	0,00	0,00	0,00	0,00

**Clinical Officer Ophthalmology**	0,00	0,06	0,17	0,00	0,05	0,33	0,11	0,18	0,31

**Clinical Officer TB, HIV/AIDS**	0,00	0,00	0,00	0,00	0,00	0,00	0,00	0,09	0,00

**Clinical Officer Psychiatry**	0,18	0,06	1,00	0,00	0,05	0,00	0,11	0,27	0,00

**Consultant**	0,18	0,45	0,50	0,18	0,77	0,00	0,00	0,18	0,08

**Consultant Anaesthesia**	0,00	0,03	0,00	0,00	0,00	0,33	0,00	0,00	0,00

**Consultant Obstetrics and Gynaecologist**	0,09	0,00	0,00	0,09	0,00	0,33	0,00	0,09	0,00

**Consultant Ophthalmology**	0,00	0,03	0,00	0,00	0,00	0,00	0,00	0,00	0,00

**Consultant Surgeon**	0,00	0,03	0,00	0,00	0,00	0,00	0,00	0,00	0,00

**Dental Surgeon**	0,09	0,10	0,17	0,00	0,07	0,33	0,11	0,00	0,08

**Dental Technician**	0,09	0,00	0,00	0,00	0,00	0,00	0,00	0,00	0,00

**Dental Technologist**	0,18	0,19	0,83	0,09	0,05	0,33	0,11	0,45	0,08

**Dental Therapist**	1,27	0,94	1,67	0,36	0,56	1,33	0,89	1,55	0,54

**Environmental Health Expert**	0,36	0,35	0,50	0,36	0,05	2,00	0,67	0,73	0,08

**Environmental Health Officer**	0,36	0,19	1,33	0,45	0,13	0,67	1,00	0,73	0,38

**Environmental Health Technologist**	8,00	2,94	17,33	3,82	1,39	17,33	9,56	15,09	6,85

**Laboratory Technologist**	1,82	1,52	2,00	1,00	0,61	3,00	1,33	2,45	0,69

**Laboratory Technician**	1,55	1,87	2,50	1,00	0,59	3,33	1,56	2,64	0,77

**Medical Licentiate**	0,18	0,03	1,00	0,18	0,07	0,33	0,33	0,09	0,23

**Nutritionist**	0,45	0,29	1,50	0,18	0,26	0,67	0,56	0,82	0,15

**Occupational Health Technologist**	0,00	0,00	0,00	0,00	0,00	0,00	0,00	0,09	0,00

**Occupational Therapist**	0,00	0,00	0,00	0,00	0,00	0,00	0,00	0,09	0,00

**Pharmacist**	0,45	0,39	0,50	0,09	0,15	0,00	0,33	0,64	0,15

**Pharmacy Dispenser**	2,73	2,35	3,00	0,73	1,02	2,00	1,78	4,00	1,62

**Pharmacy Technician**	0,18	0,19	0,17	0,27	0,00	0,67	0,11	0,09	0,15

**Pharmacy Technologist**	0,45	0,68	2,00	0,55	0,36	1,33	0,44	1,36	0,15

**Physiotherapist**	1,55	1,29	1,33	0,45	0,57	1,67	0,89	1,82	0,62

**Physiotherapy Technologist**	0,09	0,06	0,67	0,27	0,03	0,00	0,33	0,64	0,08

**Public Health Nurse**	0,18	0,06	0,17	0,00	0,13	0,33	0,11	0,00	0,23

**Radiographer**	1,27	1,26	2,00	0,36	0,59	1,33	1,11	2,55	0,08

**Radiography Technologist**	0,00	0,00	0,00	0,00	0,07	0,00	0,44	0,00	0,00

**Registered ICU Nurse**	0,00	0,00	0,17	0,00	0,00	0,00	0,00	0,09	0,00

**Registered Midwife**	3,45	3,13	2,67	0,55	1,66	4,67	1,78	4,27	0,92

**Registered Nurse**	9,09	10,23	15,00	4,91	5,97	13,33	13,33	12,91	3,54

**Registered Psychiatry Nurse**	0,18	0,03	0,00	0,00	0,25	0,00	0,00	0,18	0,00

**Registered Theatre Nurse**	0,45	0,35	0,83	0,36	0,30	0,33	0,44	0,64	0,15

**Registrar**	0,09	0,77	0,17	0,27	0,74	0,00	0,00	0,45	0,00

**Registered Nurse Ophthalmology**	0,00	0,00	0,00	0,00	0,02	0,00	0,00	0,00	0,00

**Resident Medical Officer**	1,18	2,32	2,67	0,82	1,44	5,00	1,56	3,27	0,85

**Zambia Enrolled Midwife**	16,00	12,26	29,83	4,91	4,95	14,67	15,78	36,18	6,08

**Zambia Enrolled Nurse**	51,09	33,68	76,50	25,45	16,79	115,67	39,22	73,55	25,15

**Zambia Enrolled Ophthalmology**	0,00	0,00	0,00	0,00	0,00	0,00	0,00	0,09	0,00

**Zambia Enrolled Psychiatry Nurse**	0,36	0,26	0,33	0,45	0,67	0,00	0,11	0,09	0,15

**Zambia Enrolled Theatre Nurse**	0,27	0,23	1,50	0,18	0,05	0,00	0,44	0,45	0,23

**Other**	47,00	73,74	192,83	47,91	46,36	183,33	132,44	132,64	51,92

Source: MoH, March 2008 payroll data base

There is a similar uneven distribution in the ratio of population per cadre (Table 3). For the 52 cadres listed, the best served provinces, were Copperbelt (13 cadres with a ratio above the national median), Southern (16), Lusaka (19), Central (23), Western (33 cadres), North-Western (36), Eastern (38), Luapula (40) and Northern (43).

**Table 3:** Population per health worker per province, Zambia, 2008

Position	Central	Copperbelt	Eastern	Luapula	Lusaka	N Western	Northern	Southern	Western
	
	ratio	ratio	ratio	ratio	ratio	ratio	ratio	ratio	ratio
**General Medical Officer**	111648	53933	249819	87875	29019	237528	173177	129206	66681

**Anaesthesiologist**	-	-	-	966624	-	-	-	-	-

**Biomedical Scientist**	1228125	334385	-	-	84293	-	1558589	355318	-

**Clinical Officer**	11478	7776	15946	19727	7696	12286	16581	7641	11258

**Clinical Officer Anaesthesia**	245625	238846	499637	322208	177016	237528	311718	109328	866854

**Clinical Officer Dental**	614063	835962	1498912	-	442540	-	-	473757	866854

**Clinical Officer Dermatology**	1228125	835962	-	-	-	356293	1558589	-	866854

**Clinical Officer Eye, Nose, and Throat**	-	1671923	-	-	-	-	-	-	-

**Clinical Officer Ophthalmology**	-	835962	1498912	-	590053	712585	1558589	710635	216714

**Clinical Officer TB, HIV/AIDS**	-	-	-	-	-	-	-	1421270	-

**clinical Officer Psychiatry**	614063	835962	249819	-	590053	-	1558589	473757	-

**Consultant**	614063	119423	499637	483312	37663	-	-	710635	866854

**Consultant Anaesthesia**	-	1671923	-	-	-	712585	-	-	-

**Consultant Obstetrics and Gynaecologist**	1228125	-	-	966624	-	712585	-	1421270	-

**Consultant Ophthalmology**	-	1671923	-	-	-	-	-	-	-

**Consultant Surgeon**	-	1671923	-	-	-	-	-	-	-

**Dental Surgeon**	1228125	557308	1498912	-	442540	712585	1558589	-	866854

**Dental Technician**	1228125	-	-	-	-	-	-	-	-

**Dental Technologist**	614063	278654	299782	966624	590053	712585	1558589	284254	866854

**Dental Therapist**	87723	57653	149891	241656	52064	178146	194824	83604	123836

**Environmental Health Expert**	307031	151993	499637	241656	590053	118764	259765	177659	866854

**Environmental Health Officer**	307031	278654	187364	193325	221270	356293	173177	177659	173371

**Environmental Health Technologist**	13956	18373	14413	23015	20825	13704	18123	8562	9740

**Laboratory Technologist**	61406	35573	124909	87875	47842	79176	129882	52640	96317

**Laboratory Technician**	72243	28826	99927	87875	49171	71259	111328	49009	86685

**Medical Licentiate**	614063	1671923	249819	483312	442540	712585	519530	1421270	288951

**Nutritionist**	245625	185769	166546	483312	110635	356293	311718	157919	433427

**Occupational Health Technologist**	-	-	-	-	-	-	-	1421270	-

**Occupational Therapist**	-	-	-	-	-	-	-	1421270	-

**Pharmacist**	245625	139327	499637	966624	196684	-	519530	203039	433427

**Pharmacy Dispenser**	40938	22903	83273	120828	28551	118764	97412	32302	41279

**Pharmacy Technician**	614063	278654	1498912	322208	-	356293	1558589	1421270	433427

**Pharmacy Technologist**	245625	79615	124909	161104	80462	178146	389647	94751	433427

**Physiotherapist**	72243	41798	187364	193325	50576	142517	194824	71064	108357

**Physiotherapy Technologist**	1228125	835962	374728	322208	885080	-	519530	203039	866854

**Public Health Nurse**	614063	835962	1498912	-	221270	712585	1558589	-	288951

**Radiographer**	87723	42870	124909	241656	49171	178146	155859	50760	866854

**Radiography Technologist**	-	-	-	-	442540	-	389647	-	-

**Registered ICU Nurse**	-	-	1498912	-	-	-	-	1421270	-

**Registered Midwife**	32319	17236	93682	161104	17526	50899	97412	30240	72238

**Registered Nurse**	12281	5274	16655	17900	4863	17815	12988	10009	18845

**Registered Psychiatry Nurse**	614063	1671923	-	-	118011	-	-	710635	-

**Registered Theatre Nurse**	245625	151993	299782	241656	98342	712585	389647	203039	433427

**Registrar**	1228125	69663	1498912	322208	39337	-	-	284254	-

**Registered Nurse Ophthalmology**	-	-	-	-	1770159	-	-	-	-

**Resident Medical Officer**	94471	23221	93682	107403	20115	47506	111328	39480	78805

**Zambia Enrolled Midwife**	6978	4400	8374	17900	5861	16195	10976	3571	10973

**Zambia Enrolled Nurse**	2185	1601	3266	3452	1729	2054	4415	1757	2651

**Zambia Enrolled Ophthalmology**	-	-	-	-	-	-	-	1421270	-

**Zambia Enrolled Psychiatry Nurse**	307031	208990	749456	193325	43175	-	1558589	1421270	433427

**Zambia Enrolled Theatre Nurse**	409375	238846	166546	483312	590053	-	389647	284254	288951

**General support staff**	2375	731	1296	1834	626	1296	1308	974	1284

Sources: population figures were obtained from the Zambia 2000 "census of population and housing", and extrapolated using expected growth rates for each province; for health workers the source was the MoH, March 2008 payroll database

The provincial distribution of health specific occupations showed a skewed staff distribution in favour of the most urbanized provinces (Lusaka and Copperbelt provinces). The Zambia enrolled nurse is the occupation with the most uniform distribution across provinces.

#### Distribution of staff by levels of care

Non-qualified health workers (ancillary staff) constituted the greatest majority of workers at Level 3 hospitals, followed by enrolled nurses and registered nurses. There were no consultant surgeons, anaesthetists, laboratory and radiology staff. For 50 occupational categories in the two provinces with Level 3 hospitals (Copperbelt and Lusaka), staffing levels were below the approved establishment for 26 and 35 categories respectively, above for 14 and 9 categories, and equal for 10 and 6 categories.

General medical officers represented between 0.3% and 2.6% of the workforce for Level 2 hospitals per province; clinical officers between 2% and 4%; registered nurses between 4% and 8%; enrolled nurses between 16% and 28% and general nonqualified workers between 41% and 57%. The ratio of non-qualified workers to general medical officer varied from 19 to 137. The ratio of all cadres per bed was generally low, and more so for general medical officers per 100 beds at between 0 and 4.

For Level 1 hospitals the situation was similar. General medical officers represented between 0.3% to 2.9% of the total workforce; clinical officers between 2% to 6%; registered nurses between 4% to 8%; enrolled nurses between 17% to 34% and general non-qualified workers between 34% to 53%; ratios to general medical officer varied from 16 to 159. In Level 1 hospitals, the ratio of cadres per bed was also low: the ratio of general medical officers per 100 beds varied between 0 and 3.

Only two physicians worked in rural health centres in the whole country. Placing doctors at this level may be questionable, but some large health centres function as first level hospitals without being categorized as such by the MoH, and would therefore justify employing physicians. Non-qualified workers formed between 31% to 54% of all staff in rural health centres; clinical officers between 3% and 11%; enrolled midwives between 3% and 14%; environmental health technologist between 8% and 15%; and enrolled nurses between 16% and 27%. The ratio of non-qualified workers to clinical officer varied between 3 and 16 per province.

Urban health centres employed 17 doctors. These facilities also often functioned as first level hospitals, especially in Lusaka which had only tertiary hospitals. The infrastructure of urban health centres was upgraded to enable them to function at a higher level of service provision. Non-qualified workers constitute between 17% to 33% of total staff; clinical officers between 4% and 11%; enrolled midwives between 9% and 2%; environmental health technologist between 0% and 7%; and enrolled nurses between 22% and 45%. The ratio of non-qualified workers to clinical officer varied between 2 and 8 by province.

In the entire country, the data set reported a total 109 staff in health posts: 1 clinical officer, 3 environmental health technologists, 2 registered nurses, 12 enrolled midwives, 32 enrolled nurses, and 59 others.

#### Vacancy rates

For Level 3 facilities, vacancy rates varied between 38% in the Copperbelt Province and 5% in the Lusaka Province; for Level 2 facilities, figures were 30% and 70% in the Western and Copperbelt provinces; for Level 1 facilities, 54% and 80% for the Southern and Western provinces. For rural health centres, rates varied between 15% and 63% (Lusaka and Luapula) and for urban health centres between 13% for Lusaka and 96% for the Western provinces.

## Discussion: explaining the observed shortages and maldistribution

Zambia enrolled nurses are the most prevalent health specific cadre. This cannot be ignored in any policy to correct the impact of shortages and imbalances.

We identified a severe shortage, reflected in high vacancy rates of personnel in Zambia, associated with imbalances between provinces, levels of care and in the mix of cadres.

Shortages might be due to the inability to compensate for attrition and growing needs with scaled-up production, recruitment and retention of health workers.

High vacancy rates have been described in other studies: Picazo and Kagulura report that the percentage of vacant posts in 2005/2006 was 42% in rural health centres, 22% in urban health centres, and 41% in hospitals (or 33.6% overall). Key posts left vacant all involved professional staff. Districts with high rates of vacancy (> 50%) among professional staff included: Chilubi, 79%; Chinsali, 58%; Kalomo, 59%; Kasama, 66%; Mpika, 57%; Mpongwe, 53%; Mufulira, 66%; Nakonde, 60%; Namwala, 54%; Sesheka, 74%; Shangombo, 56% [9].

Geographical imbalances of personnel can be attributable to a number of factors [10], of which we identify some below.

### Health workforce policies

The Zambian health sector has shown capacity for HRH innovation. Examples are initiatives such as upgrading the level of training (new degree courses launched or projected, e.g. BSc Nursing), facilitating direct access to diploma level specialist training (e.g. clinical officer, psychiatry, midwifery and mental health nursing), creating new cadres to formalize task delegation from higher level cadres (e.g. dispensers, counsellors and licenciates), informal task shifting (in early 2001, the Zambian law was amended to authorize nurses to prescribe and to insert drips [11]). There were efforts to identify tasks required to meet needs and to adapt training programs to include them; an example is that of training clinical care specialists, who are physicians who receive further training to assume clinical management functions and to provide hands-on supervision to front line workers (Director HRH Administration, MoH, personal communication, May 2008). However some of the new occupations are not recognised by professional councils, e.g. dressers, care givers, psychosocial counsellors, dispensers, medical technologists. Direct entry to advanced training reduces the back-to-school attrition associated with the loss of personnel who leave their post to train and often do not return to the public sector [4].

Although Zambia trains generalist cadres with internationally recognized degrees (e.g. doctors and registered nurses), other cadres are only recognized locally or regionally (e.g. Zambia enrolled nurses, clinical officers, clinical licentiates). An example of the policy of training cadres only recognized locally or regionally is that of clinical licenciates. In 2002, the MoH initiated a two-year programme of retraining clinical officers with three years of experience or more, to the level of clinical licentiates, capacitating them with surgical and obstetric skills, and more advanced skills in paediatrics and internal medicine. This training prepares them for operating autonomously in rural hospitals or in large health centres where there are no doctors. They spend six months at the Faculty, do clinical training for 20 months, and then return for 1 month to write exams. After completion, they serve a 1 year internship. These locally recognised cadres are either substitutes or assistants to other cadres; there is a long tradition of these in Zambia that predates independence (Head, Department of Community Medicine, personal communication May 2008). They are a sort of insurance against the loss of medical skills to emigration. The same reasoning applies to two categories of nurses: Registered professional nurses, internationally recognised, have higher emigration rates than enrolled nurses, who are only locally recognized [12].

In spite of these innovations, there is a shortage of skilled nurses who assume roles not only as providers of nursing care, but also as substitute to other health workers. This creates tensions which do not always help to achieve the best mix of nursing cadres and leads to misunderstandings that lead to the failure and lack of continuity of many training initiatives, such as the two year training of Zambia enrolled nurses or the six month training of nurse assistants, which were proposed by the MoH but opposed by the Union (President of Zambian Union of Nursing, personal communication May 2008).

### Emigration

The emigration of physicians and nurses is part of the general emigration of qualified workers, which in the early 2000's was estimated at 10% of qualified workers in Zambia, 16% in Tanzania, 26% in Angola, 36% in Madagascar, 42% in Mozambique, and 48% in Mauritius. For nurses, the rate was 9.2% in Zambia, 12% in Angola, 17% in Malawi, 19% in Mozambique 24% in Zimbabwe, 28%% in Madagascar, and 63% in Mauritius. It is for physicians that the rate of emigration is particularly high: 57% in Zambia's, surpassed only by Malawi (59%), Angola (71%) and Mozambique (75%) [13].

The driving forces for migration to other countries and exit of the public sector are many: low remuneration, poor working conditions, absence of career development mechanisms, civil strife and political instability, fear to contract diseases such as HIV/AIDS and policies that encourage labour export like in the Philippines [14]. Some of these factors are present in Zambia, and may explain why out of 1,200 doctors trained in Zambia since the late 1960s, only 391 are still practicing in the Zambian public sector, a decrease that cannot be explained by normal attrition resulting from retirement or death [15]. A 2006 survey of 50 health staff in Lusaka province identified different reasons for potential migration. Low salaries are an important factor driving nurses and clinical officers to look for better paying jobs outside the public sector. Salary had less importance for doctors than inadequate diagnostic equipment and supplies. Work overload and long working hours due to shortage of health staff were also identified as push factors [15].

Emigration of health workers from Zambia is partly financed by Zambia's policy of offering Voluntary Separation Packages: these are early retirement lump sum payments promoted by the government, which are used towards migration costs [12].

### Working conditions and HRH management

Working conditions are important for motivating health workers to be productive and to meet quality standards. Huddart et al [11] reported survey results showing that 100% of doctors, 80% of nurses and 92% of clinical officers wanted improvements in the cleanliness and maintenance of public health facilities. All categories of staff identified poor management of human resources as a contributory factor to issues of leave, accommodation and communication not being appropriately dealt with. This probably contributes to the high attrition rates of health personnel observed in the Zambian health sector.

This situation can be changed without significant costs as staff in the Zambian public health sector respond positively to performance-based awards [16]. Staff motivation improves substantially with even small gestures of support and encouragement from district supervisors. For example, non-financial awards are as motivating, if not more, than financial awards and do not generate as much conflict, suspicion, or frustration. Staff also feels encouraged by knowing that their performance is monitored and that this served to target support responding to their actual needs. District managers suggest that a performance-based award program, linked to the district performance management system, helps to guide them in their work, provides direction for supervisory visits and assists in monitoring health facility and district performance [16]. Many health workers generate complementary income by other activities; this is more common in urban than in rural settings (32% and 9% of staff respectively) [17]. Besides the amount of income, other considerations are important, namely the issue of regularity of payment. In the Zambian public sector up to 15% of staff do not always receive their salary and 80% complained of late payments; and 10% of staff had to pay a so-called *expediter fee *to obtain their salaries [17].

### Attrition rates

Attrition rates are also an important cause of health workforce imbalances [4]. In 2003, physicians had the highest attrition rate (9.8%) followed by nurses (5.3%), pharmacists (4.6%) and laboratory technicians (3.5%) [2]; there are differences in urban and rural attrition rates, leading to net losses of staff in rural areas (Eastern, Luapula, Northern, and Western provinces) and net staff gains in the most urbanized areas (Copperbelt and Lusaka provinces) [17]. Reasons for health personnel attrition include death (38%), resignation (32%), dismissal (12%), retirement (10%) or end of contract (8%) [5]. Feeley et al (2004) [18] estimated the cost to the public healthcare system of personnel losses due to HIV/AIDS. If transfers to other public sector institutions are excluded, the annual loss of health professionals at the sites studied was 217 out of 2,333 filled positions, an annual attrition rate of 9%. Excluding transfers, losses accounted for 21% of physician vacancies, 14% of clinical officer vacancies and 16% of nurse vacancies. In the 12 months ending in October 2003, deaths represented a significant proportion of all terminations (over 20%); death rates were 0.4% for doctors, 2.8% for clinical officers and 3.5% for nurses working at the study sites. For doctors, mortality is not the main reason for attrition, nor the most important factor contributing for the high vacancy rates. But for clinical officers and nurses, death is the single largest reason for loss. The average age at death for all health professionals was 37.7 years. The graduation of clinical officers would have to increase 80% and of nurses 50% to offset observed mortality. In the final year of employment, those who were absent because of illness recorded an average of 28 additional days of leave. Costs associated with this additional leave, plus death or retirement related payments averaged $4,056 for a doctor, $2,678 for a clinical officer and $3,674 for a nurse. In the short to medium term, the reduction of attrition and vacancies must take into account the effective treatment of AIDS, which is an important cause of absenteeism, burnout, illness and death [19-22].

### Health services system factors

Over the years, there has been a steady growth of the private healthcare sector, resulting into various forms of private-public partnerships, which include the sharing of medical equipment and technologies, referral of patients, human resources and facilities.

Right-sizing of the public sector facilities and the gradual increase of "for-profit" and "not-for-profit" private health service providers presents significant policy implications in terms of their involvement in the delivery of public health services. The private sector is a major source of drain of health workers from the public sector, particularly of laboratory personnel, pharmacists and doctors (Director HRH Administration, MoH, personal communication, May2008). Private salaries are more than double government ones for physicians, triple for laboratory technicians, and one third higher for midwives. NGOs are paying between 23% and 46% more than the government [23].

With the exception of the Churches Health Association of Zambia (CHAZ), other private sector participation in health service delivery in Zambia has been modest. CHAZ is an umbrella Christian NGO that supports 135 member institutions (hospitals, rural health centres, and community-based initiatives). It complements government efforts in the delivery of health care. A memorandum of understanding states that the MoH is mandated to provide CHAZ with trained HRH, and to pay their salary. The MoH deploys the staff, through the provincial health authorities. Issues of planning and performance assessments are dealt with the District Health Offices. The MoH also provides operational funds according to the number of beds. CHAZ provides infrastructure (according to the prototypes defined by the Government), complements Government efforts in logistics, provides expatriate HRH, and assumes the management of operations according to Government standards (Director HRH, CHAZ, personal communication, May.2008)

## Conclusion

Factors associated with health workers shortages and with imbalances in their distribution are many and reflect local and global factors that together constitute a trap that perpetuates the situation. No single measure will correct this state of affairs [5]. Measures taken must be multifaceted, and not only sector wide, but also society wide, and supported by national and international stakeholders.

Strategies to address human resources shortages like those described here, as well as unmet needs as there exist in most African countries, have centred around staff retention through incentives such as allowances, salary top-ups, and better working conditions [24], training and retraining, including shifting as many tasks as possible away from doctors, nurses and pharmacists to non-clinical staff, enabling clinical staff to concentrate on the most complex of their specific areas of expertise [25]. These strategies are in line with the 2010 WHO recommendations on increasing access to health workers in underserved areas [26]. Scaling-up the production of health workers remains a priority, but it is expensive [5,27]. The costs of addressing shortages and deficits could reduce if rates of retention, graduation and public sector entry came closer to 100% [5]. Redistributing tasks among health worker teams through broader delegation (task-shifting), can increase technical efficiency, while maintaining quality, and thereby improve access and affordability [28]. A projection of enrolment needs up to 2018 concluded that even by training more qualified staff, Zambia would still face shortages, if no other efficiency enhancing measures were taken [4]. The review of the distribution of tasks among the various health cadres is a context-specific process, because it has to be locally relevant and sensitive to potential resistance; actors such as professional councils need to be brought in into the process to make task-shifting acceptable. The demonstration of regional variations in the distribution of the health workforce is a strong argument in favour of discussing options to improve access to services, including through a strategy of task-shifting and creation of new cadres. Whose tasks will be transferred to or shared with whom has to be negotiated and decided locally, within the context of a policy aimed at improving access to health services and to reducing unmet needs.

## Competing interests

The authors declare that they have no competing interests.

## Authors' contributions

PF, SS and FG participated in the conception of the study, participated in field work, and in the writing up of the paper. SS and PF were responsible for the data analysis. GD was a consultant for the study and was involved with the writing up. All the authors read and approved the final manuscript.
